# Gene expression profiling of orbital muscles in treatment-resistant ophthalmoplegic myasthenia gravis

**DOI:** 10.1186/s13023-020-01629-9

**Published:** 2020-12-11

**Authors:** Tarin A. Europa, Melissa Nel, Jeannine M. Heckmann

**Affiliations:** 1 Neurology Research Group, Division of Neurology, Department of Medicine, Faculty of Health Sciences, E8-74, New Groote Schuur Hospital, University of Cape Town, Cape Town, 7925 South Africa; 2 UCT Neuroscience Institute, Cape Town, South Africa

**Keywords:** Orbicularis oculi, Muscle atrophy, Oxidative metabolism, Ophthalmoplegia, Extraocular muscles, Contractility, Myasthenia gravis, Treatment-resistant, Gene expression

## Abstract

**Background:**

Unbiased in silico approaches applied to genome-wide data prioritized putative functional gene variants associating with treatment-resistant ophthalmoplegic myasthenia gravis (OP-MG). Although altered expression of genes harbouring these variants, or associated pathways, were shown in patient-derived transdifferentiated-myocyte models, gene expression in orbital-derived muscle was required to test the validity of the predictions.

**Methods:**

We sampled orbicularis oculi muscle (OOM) and one paralysed extraocular muscle (EOM) from six individuals with OP-MG during blepharoptosis and re-alignment surgeries, respectively. For controls, the OOMs were sampled from four individuals without myasthenia undergoing surgery for non-muscle causes of ptosis, and one non-paralysed EOM. Using a qPCR array, expression of 120 genes was compared between OP-MG and control OOMs, profiling putative “OP-MG” genes, genes in related biological pathways and genes reported to be dysregulated in MG cases or experimental MG models, and in EOMs of cases with strabismus. Normalization was performed with two stable reference genes. Differential gene expression was compared between OP-MG and control samples using the ΔΔCT method. Co-expression was analysed by pairwise correlation of gene transcripts to infer expression networks.

**Results:**

Overall, transcript levels were similar in OOMs and EOMs (*p* = 0.72). In OOMs, significant downregulated expression of eight genes was observed in OP-MG cases compared with controls (> twofold; *p* ≤ 0.016), including *TFAM*, a mitochondrial transcription factor, and genes related to the following pathways: atrophy signalling; muscle regeneration and contraction; glycogen synthesis; and extracellular matrix remodelling. Several microRNAs, known to be highly expressed in EOMs, are predicted to regulate some of these genes. Co-expression analyses of gene-pairs suggested high interconnectedness of gene expression networks in OP-MG muscle, but not controls (r > 0.96, *p* < 0.01). Significant inverse directions of gene-pair correlations were noted in OP-MG versus controls OOM networks (r ≥ 0.92, *p* < 0.001) involving most OP-MG genes overlapping prominently with muscle atrophy/contractility and oxidative metabolism genes.

**Conclusions:**

The gene expression in orbital muscles derived from OP-MG individuals compared with normal controls, support the pathogenic hypothesis previously generated from whole genome sequence analyses. Repression of gene transcripts in OP-MG orbital muscle implicate tissue-specific regulatory mechanisms, which may inform future biomarker discovery approaches.

## Introduction

Previously we identified individuals with otherwise characteristic myasthenia gravis (MG) and who responded to immune therapies as expected in their non-ocular muscles, but who remained with treatment-resistant ophthalmoplegia with/without ptosis [1, 2]. These individuals are most frequently MG subjects with juvenile onset symptoms, African genetic ancestry (see methods) and who have generalised disease with detectable circulating acetylcholine receptor (AChR) antibodies [1, 3]. Clinically, the treatment-resistant weakness of the extraocular muscles (EOMs) may range from moderate to severe. When severe, in which case most of the EOMs are paralysed, we refer to the subphenotype of treatment-resistant ophthalmoplegic MG, or OP-MG. In an African setting we anticipate up to 20% of cases may develop treatment-resistant EOM weakness [4].

To understand the underlying genetic pathogenesis of the OP-MG subphenotype we previously used next generation sequencing to dissect the molecular genetic landscape using an extreme phenotype approach i.e. OP-MG cases vs control MG. Genes associated with OP-MG were identified using single variant and gene-based cumulative variant statistical association analyses [5, 6]. The pathogenetic pathways postulated to be altered in OP-MG, and supported by expression studies in patient-specific dermal-derived transdifferentiated myocyte models, related to muscle regeneration and atrophy signalling [7]. The aim of this work was to investigate the expression of these putative OP-MG susceptibility genes/pathways in tissues relevant to the subphenotype, i.e. patient-derived orbital muscles, and to verify the hypotheses generated by the unbiased genomic studies in the tissues of OP-MG cases vs controls without MG.

**Fig. 1:**
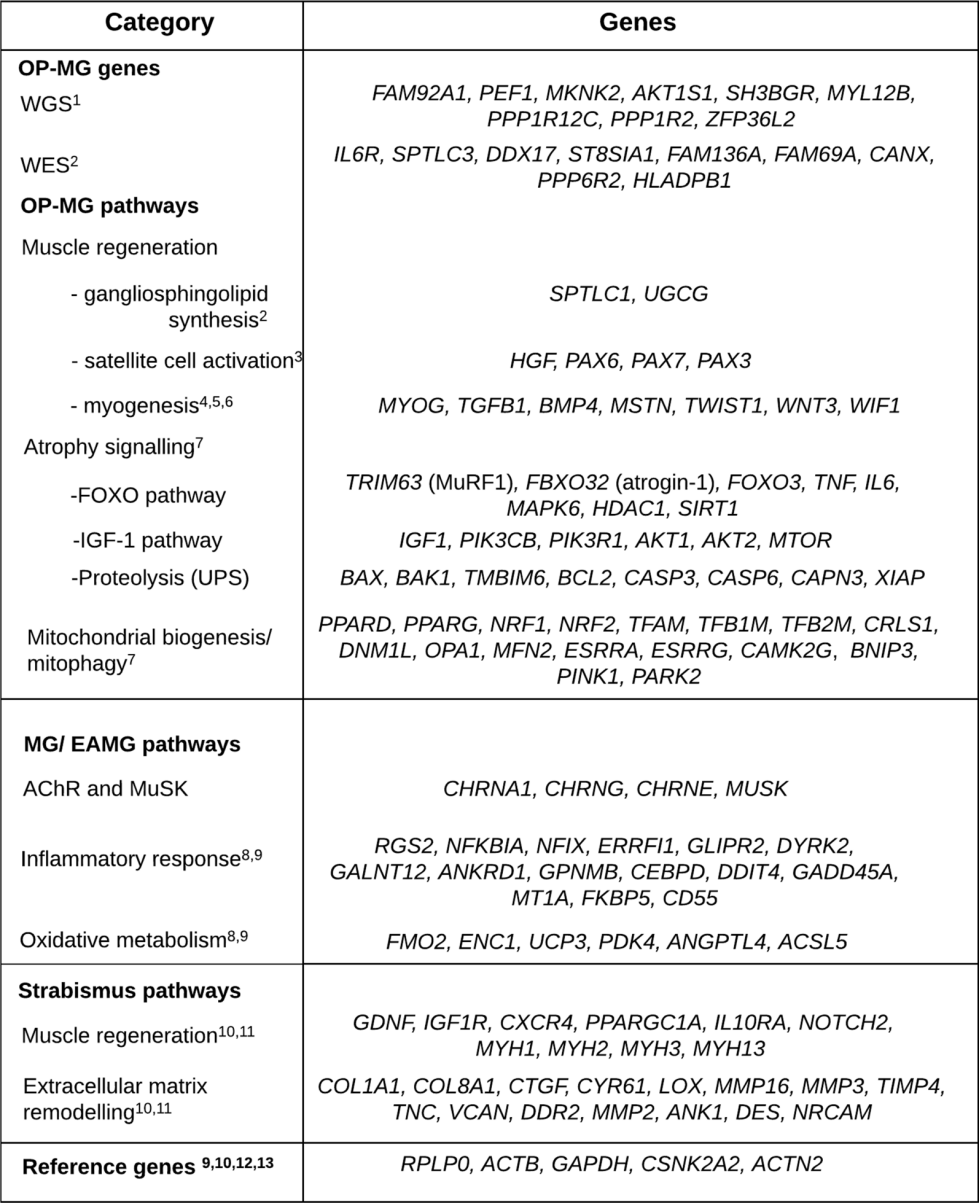
Genes profiled in the array organised by category. Ophthalmoplegic myasthenia gravis (OP-MG). “OP-MG genes” refers to genes identified by unbiased next generation sequencing analyses in OP-MG and control-MG subjects, either by whole genome sequencing (WGS) or whole exome sequencing (WES). “OP-MG pathways” refers to the main biological pathways associated with the known physiological functions of the putative OP-MG genes. The “atrophy signalling” genes were selected from biological pathways in reference 7 using associated KEGG pathways as resources. Ubiquitin proteasome system (UPS); experimental autoimmune myasthenia gravis (EAMG); acetylcholine receptor (AChR); muscle-specific kinase (MuSK); extracellular matrix (ECM). “Strabismus pathways” refers to genes with altered expression in human strabismic extraocular muscles. References in figure: (1) [5]; (2) [6]; (3) [50]; (4) [51]; (5) [12]; (6) [52]; (7) [32]; (8) [53]; (9) [18]; (10) [21]; (11) [22]; (12) [7]; (13) [54]

Briefly, a genome-wide single nucleotide variant analysis followed by prioritization based on skeletal muscle gene expression (from GTExPortal) [8], identified putative modifier variants in *FAM92A1* and *PEF1*, which were more frequent in OP-MG vs control MG genomes (*p* < 1 × 10^–5^) [6]. As an adjunct, we sought genes with a cumulative variant burden at gene-level associating with OP-MG compared to control MG genomes, and after filtering by relevance in muscle (GTExPortal), identified several genes which may be relevant in OP-MG pathogenesis [6]. For simplicity we will refer to genes from both single-variant and gene-based analyses as putative “OP-MG genes” (Fig. 1, OP-MG_WGS_ genes). Although the aim of the limb muscle-based prioritization was to select genes with biological relevance in this tissue, a limitation of this approach was that gene expression in EOM, as an unique muscle group, was not specifically considered due to the limited availability of gene expression data.

The EOMs constitute a unique muscle allotype [9–11]. However, we have used biopsies of orbicularis oculi muscle (OOM) as a surrogate orbital muscle to profile transcripts which may closely resemble the EOM transcriptome. We have done so for several reasons; the relative inaccessibility of EOMs; the ultra-rare indications to offer oculoplastic surgery in MG and therefore the possibility of EOM biopsy; and the paralysed EOMs in OP-MG would reflect the gene expression profile of this ‘burnt-out’ stage which may differ from the active evolving pathophysiological process.

As craniofacial muscles, both EOMs and OOMs share similar embryological origins and many of the anatomical, physiological and molecular characteristics which distinguish EOMs from skeletal limb muscle [12]. Both EOMs and OOMs have fast firing rates [13], high mitochondrial content and oxidative capacities [14, 15] and type II fibre predominance [9, 12]. Therefore, in contrast to previous work using transdifferentiated cell models which originated from dermal fibroblasts, the use of OOMs as a surrogate for EOM will provide important validation of gene expression tissue-specificity of the craniofacial myotranscriptome in OP-MG vs normal controls.

## Methods

### Patient selection and tissue collection

OP-MG cases and controls selected for ocular re-alignment or lid elevation procedures were invited to participate and provided biopsy specimens with informed consent. The diagnostic criteria of OP-MG cases have been previously described [1, 6, 7], and those included in this report all had circulating anti-acetylcholine receptor (AChR) antibodies (> 0.4 nmol/L) (Table 1). Importantly, for OP-MG patients to be eligible for ptosis surgeries (lid elevation), OOM contractility had to be sufficient to minimize the risk of post-operative corneal exposure. In contrast, the EOMs of the OP-MG case had been paralysed for several years by the time the medial rectus was sampled. Control samples were derived from cases with strabismus or ptosis from non-MG causes. All subjects had self-reported African-genetic ancestry i.e. either black African genetic ancestry, or mixed-African ancestry comprising predominantly KhoiSan ancestry, with lesser genetic contributions from black Africans, Europeans and Asians (see [6]). The study was approved by the institutional Human Research Ethics Committee (HREC 257/2012).

**Table 1:** Clinical information of patient-derived orbicularis oculi muscle and medial recti samples

Age at surgery (years)	Orbital muscle	Diagnosis	MG duration (years)
42	OOM	OP-MG	18
15	OOM^a^	OP-MG	7
27	OOM	OP-MG	10
34	OOM	OP-MG	14
55	OOM^a^	Levator dehiscence^b^	NA
51	OOM	Levator trauma^b^	NA
56	OOM	Canalicular obstruction^b^	NA
27	EOM	OP-MG	15
77	EOM	Secondary esotropia-blind from glaucoma	NA

Myasthenia gravis (MG) duration was taken from onset of symptoms. Extraocular muscle (EOM) refers to medial rectus; ophthalmoplegic MG (OP-MG); orbicularis oculi muscle (OOM)

^a^Samples obtained from both eyes at independent surgeries

^b^Normal OOM biopsied

Biopsies of the orbital part of the orbicularis oculi (OOM) were received from those undergoing lid elevation procedures and medial recti muscle samples (EOM) from ocular realignment surgery. Both OOM and EOM samples were dissected by the ophthalmic surgeon without cautery or clamping, immediately placed in RNAlater (ThermoFisher) and stored at -80 °C.

### RNA isolation

The orbital muscle samples were homogenized in TRI Reagent (ThermoFisher) using a handheld Pellet Pestle (Fisherbrand). After chloroform precipitation, RNA was isolated from the aqueous phase using a spin-column method (Direct-zol Miniprep kit, Zymo) according to the protocol which included an in-column DNAse step. RNA was quantified using Nanodrop 1000 software (v3.5.2, Inqaba Biotechnical industries) and RNA integrity (RIN) was evaluated using the PicoChip Bioanalyzer (Agilent 2100 Bioanalyzer). The muscle samples weighed 3–26 mg yielding 5–49 ng/µL RNA. Spectrophometric ratios suggested some impurities despite optimised RNA extraction protocols using phenol/chloroform precipitation, but were in the expected range for RNA isolated from muscle [16] with A260/280 range 1.6–1.9 and A260/230 range 0.4–1.6. RIN values ranged between 5.2 and 7.6.

### Custom array plate

A total of 120 target and 5 reference genes were profiled in a custom qPCR array (Qiagen) using proprietary assays. The target genes were categorized into OP-MG genes, OP-MG pathways, MG pathways and strabismus pathways based on the source of evidence for their relationship to OP-MG, MG or strabismus, although there is overlap between categories (Fig. 1). OP-MG genes broadly refer to those identified by WGS [6] and the preceding whole exome sequencing (WES) analysis [5], although the prime objective of this study was to validate the genes identified by the WGS analysis. OP-MG genes also served as “seed queries” which informed the broader enquiry of additional gene candidates in related pathways (OP-MG pathways). Genes previously shown to be dysregulated by MG in human limb muscles [17], in vitro models or experimental autoimmune myasthenia gravis (EAMG) [18] were also included. As reduced contractility (as occurs in OP-MG EOMs) [1, 19] potentially alters the regulation of biological pathways in ocular muscle, we also profiled genes previously shown to be dysregulated in the EOMs of cases with ocular misalignment/strabismus due to other non-myasthenic causes. We postulated that functional variants in muscle contractility pathways may render EOMs vulnerable in the setting of MG weakness.

The reference genes were selected from genes that have previously been validated in human [9, 20–22] and rodent EOMs [18, 23] and showed stability in a patient derived muscle model [7].

### Reverse transcription, qPCR and normalization

For each muscle RNA sample 40 ng was reverse transcribed using the RT^2^ First Strand Kit (Qiagen) according to the manufacturer’s protocol and qPCR performed using the RT^2^ SYBR Green Mastermix (Qiagen) on the QuantStudio 12K Flex thermocycler. No inhibition of reverse transcription or PCR was evident and no genomic DNA contamination was detected. Three control EOM samples with the lowest RNA concentrations and RIN values were excluded from data analysis for quantitation cycle (Cq) values > 35 for at least 15% of genes in the array (see Additional file 1: Table S1). Reference genes for normalization were selected based on the geNorm [24] and Bestkeeper methods for OOMs [25] and chosen based on stability of expression levels for the two EOMs.

### Gene expression analysis and statistical methods

Raw Cq values from the array were analysed using the ΔΔCt method [26]. Differences between OP-MG and controls were expressed as fold change or log_2_ fold change (Prism Graphpad 8). For OP-MG vs control comparisons in OOMs, unpaired Student’s t tests for normally distributed 2^−∆Cq^ values and Mann–Whitney U tests for non-parametric data, were performed. Shapiro Wilk tests were performed to assess normality. The Benjamini–Hochberg correction was performed on the results of the t tests and Mann–Whitney U tests in the OOM comparisons. Due to the small sample, the false discovery rate (FDR) correction was set at 15%. Statistical significance of the difference in gene expression by phenotype could not be calculated for EOMs.

Using OOM data, pairwise gene co-expression by phenotype was performed by Pearson’s correlations. Genes not expressed in > 1 sample (Cq > 35) were excluded from these analyses (n = 5). Correlation plots using gene pairs with significant correlations (*p* < 0.01) were generated using the Corrplot R package. Hierarchical clustering of the OP-MG correlation plot was performed using Hmisc (R package) and the controls correlation plot was reordered to match for visual comparison. Co-expression networks were generated using Cytoscape (v.3.71). Potentially functional modules were identified using the MCODE Cytoscape plug-in. To determine which gene pairs were significantly different between OP-MG and controls, differential correlation analysis was performed using the DiffCorr R package [27]. Gene pairs with a local false discovery rate > 0.6 were excluded.

## Results

Six OP-MG muscle (1 EOM; 5 OOM) and five control muscle (1 EOM; 4 OOM) samples passed data quality analysis (Table 1).

### Differential gene expression in orbicularis oculi muscles

To assess differences in relative gene expression between 5 OP-MG and 4 control OOMs, data normalization was performed using the average values for *RPLP0* and *ACTN2* as reference genes (Additional file 1: Table S2).

**Fig. 2:**
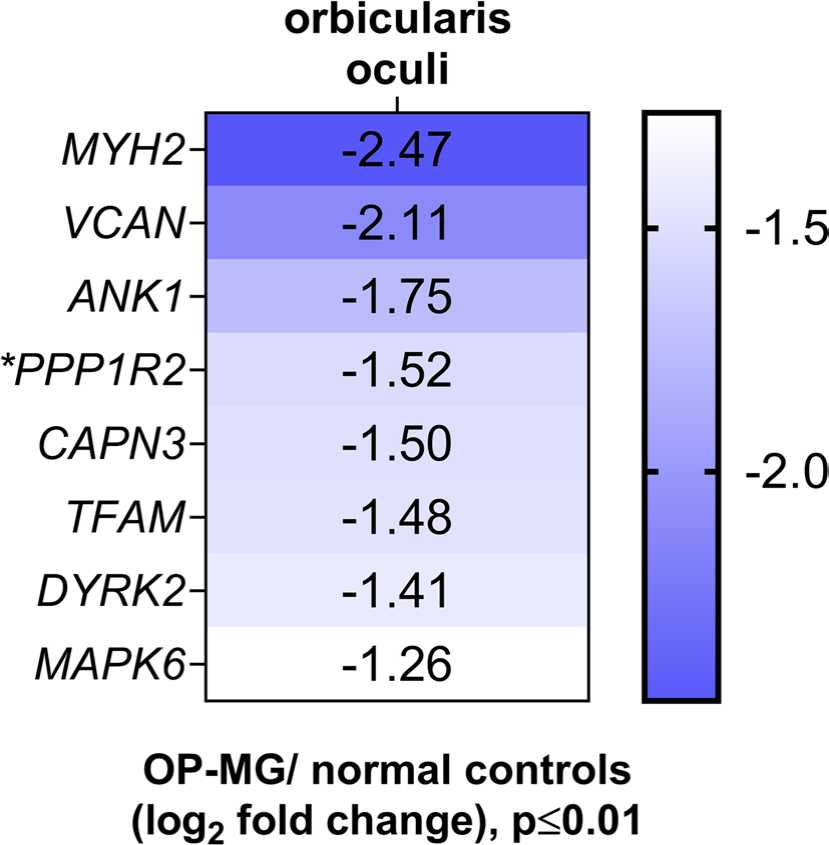
Genes differentially expressed in orbicularis oculi muscles. The differentially expressed genes between the OP-MG and control orbicularis oculi muscles represented as a heatmap (log_2_ fold change)

Figure 2 shows the genes that showed > twofold difference in gene expression levels between OP-MG vs control OOMs (FDR = 15%; *p* < 0.01). All the significantly differentially expressed genes were downregulated in the OP-MG muscles. Interestingly, the three most differentially repressed gene transcripts in the OP-MG samples were those genes included in the profiling array because of their altered expression levels reported in post-mortem strabismic EOMs compared with controls [21]. *MYH2*, which encodes the myosin heavy chain 2a isoform, found in fast-twitch muscle fibres and abundant in both OOMs [13] and EOMs [28], showed fivefold downregulation compared to controls (*p* = 0.008). *VCAN* and *ANK1*, genes related to extracellular matrix remodelling pathways, as well as genes related to muscle atrophy signalling, *CAPN3* and *MAPK6* (*p* < 0.009) were downregulated in the OP-MG OOMs. *VCAN* and *ANK1* encode components of the extracellular matrix and muscle, respectively, that regulate myoblast fusion/differentiation and attachment to the sarcoplasmic reticulum in regenerating muscle [29]. *CAPN3* (calpain 3) is involved in preserving sarcomeric integrity [30]. *MAPK6/ERK3* encodes mitogen-activated protein kinase-6, and acts at integration points for multiple processes including myogenesis [31]. *TFAM*, encoding a critical mitochondrial DNA transcription factor [32], was differentially downregulated (*p* = 0.005) in OP-MG.

*PPP1R2* which was prioritized as an OP-MG gene and showed significant repression in OP-MG orbital muscles (2.9-fold; *p* = 0.016), encodes a key enzyme in glycogen synthesis, phosphatase-1 regulatory inhibitor subunit R-2. The repression of *PPP1R2* transcripts together with *DYRK2* (2.7-fold; *p* = 0.003), which phosphorylates glycogen synthase, suggest dysregulation of muscle energy metabolism in OP-MG muscles, but not control muscle.

Overall, we found differential repression of genes involved in the maintenance of the extracellular matrix and sarcomeric stability, as well as mitochondrial biogenesis and muscle metabolism in orbital muscles from OP-MG cases vs normal controls. Interestingly, genes in the latter categories were included in the array based on postulated pathway involvement informed by genomic studies, whereas the former were considered “strabismus-pathway” genes at the time and included as a controls for the clinical phenotype of altered EOMs contractility in OP-MG.

### Differential gene-co-expression in orbicularis oculi muscle by phenotype

**Fig. 3:**
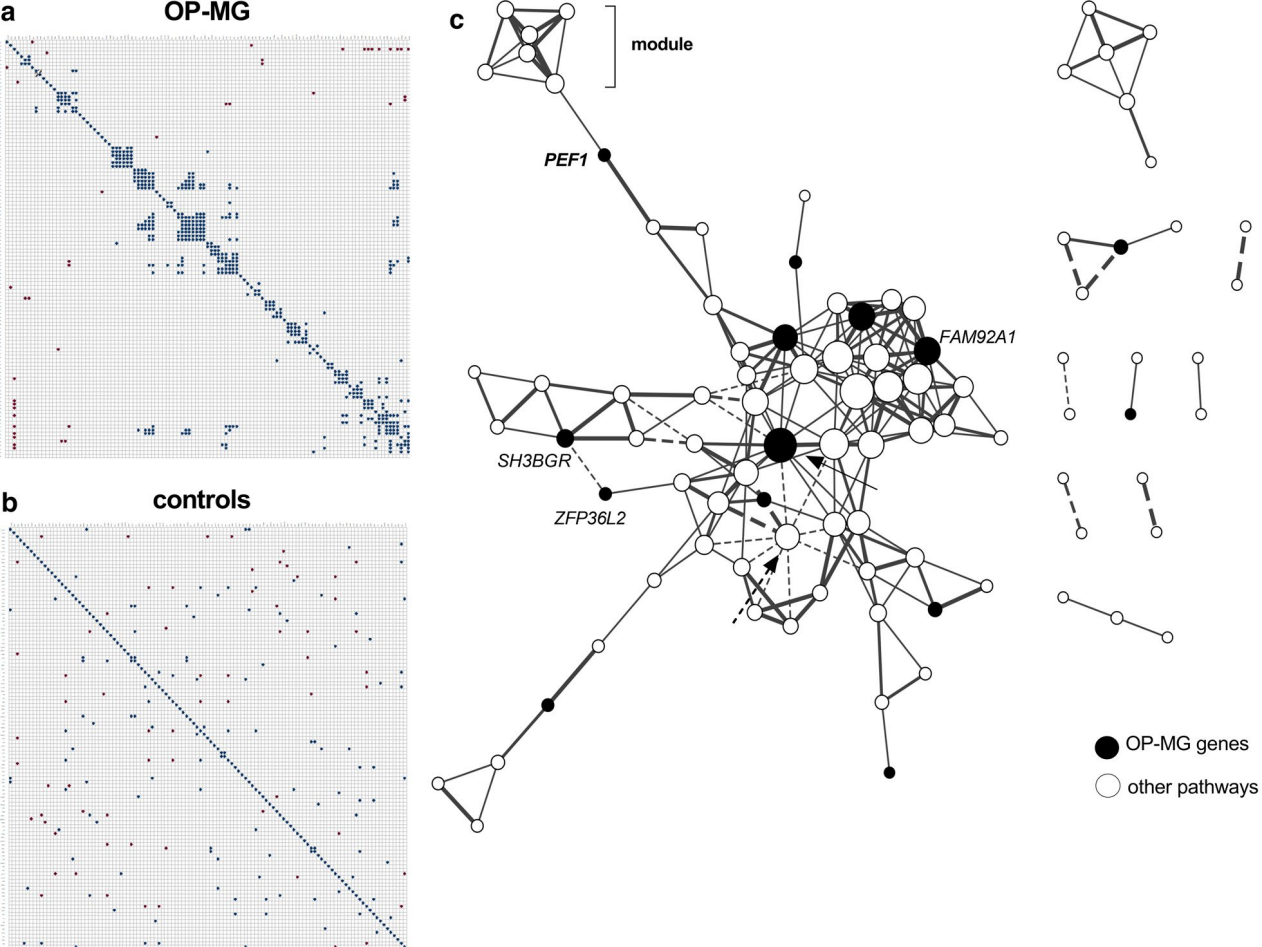
Visual comparison of gene co-expression between OP-MG and control orbicularis oculi muscles. **a** Ophthalmoplegic myasthenia gravis (OP-MG) orbicularis oculi muscles (OOMs) correlation plot with gene pairs (r > 0.96; *p* < 0.01). **b** Control correlation matrix (r > 0.98; *p* < 0.01) ordered to match hierarchical clustering configuration of A for comparison. **c** Gene co-expression network of OP-MG orbicularis oculi muscles (r > 0.96; *p* < 0.01); the OP-MG genes, identified by whole genome sequencing analysis, are labelled (intermodular gene in bold). Degree of interconnectivity is shown by increased node size. Interconnecting lines are weighted by strength of correlation. Dashed lines indicate negative correlations. The solid arrow indicates *DDX17,* which is central to a subnode and the most highly prioritized gene from the whole exome sequencing analysis [5]. The dashed arrow indicates *PARK2* which is central to an inhibitory subnode

Pairwise correlations identified 232 strongly correlated gene pairs in OP-MG OOMs and 92 gene pairs in controls (r > 0.96, *p* < 0.01). OP-MG and control correlation plots were generated using this data and hierarchical clustering (k-median) identified potentially functional modules [33] visualised in the OP-MG plot that were not seen in the control plot (Fig. 3a vs b). Figure 3c shows the visual network of co-expressed gene pairs in the OOMs of OP-MG samples (from Fig. 3a) and the most interconnected genes (10–15 gene–gene interactions each) involved putative OP-MG genes from the WGS analysis (*FAM92A1*) and from the WES analysis (*CANX*, *DDX17*, *FAM69A*) (Fig. 3c; unlabelled OP-MG genes are from the WES analysis). *DDX17* (Fig. 3c solid arrow) is a master transcriptional regulator involved in muscle gene expression [34] and appears central to a subnode. *CANX* encodes calnexin, a chaperone protein that facilitates the assembly of AChR subunits [35], *FAM92A1* has recently been recognized for its importance in mitochondrial functioning [36] and both *FAM69A* and *PEF1* have poorly characterized biological function. *PEF1* encoding Peflin, which may be involved with endoplasmic reticulum-golgi transport and/or calcium binding [37], connected with a ‘module’ of genes (Table 2) which were profiled because they showed altered muscle expression levels in EAMG [18], and they are known to regulate mitochondrial biogenesis and oxidative metabolism. *PARK2*, encoding parkin, a major regulator of mitophagy [32], appeared to form a subnode of negatively correlated genes (Fig. 3c, dashed arrow), several of which are involved in muscle regeneration (e.g. *DDX17*) and mitochondrial biogenesis. *PPP1R2*, which was differentially repressed in OP-MG muscle (Fig. 2), did not correlate with other transcripts in OP-MG muscle yet showed significant correlations with four genes in control OOMs (data not shown).

**Table 2:** Genes in putative co-expression network with PEF1 transcripts in OP-MG orbicularis oculi muscle

Gene	Function (biological pathway)
*ANGPTL4*	Oxidative metabolism
*PPARG*	Mitochondrial biogenesis/metabolism
*FMO2*	Oxidative metabolism
*BCL2*	Mitochondrial biogenesis/metabolism
*TIMP4*	Inhibitor of metalloproteinases (extracellular matrix remodelling)
*MMP16*	Metalloproteinase (extracellular matrix remodelling)

**Fig. 4:**
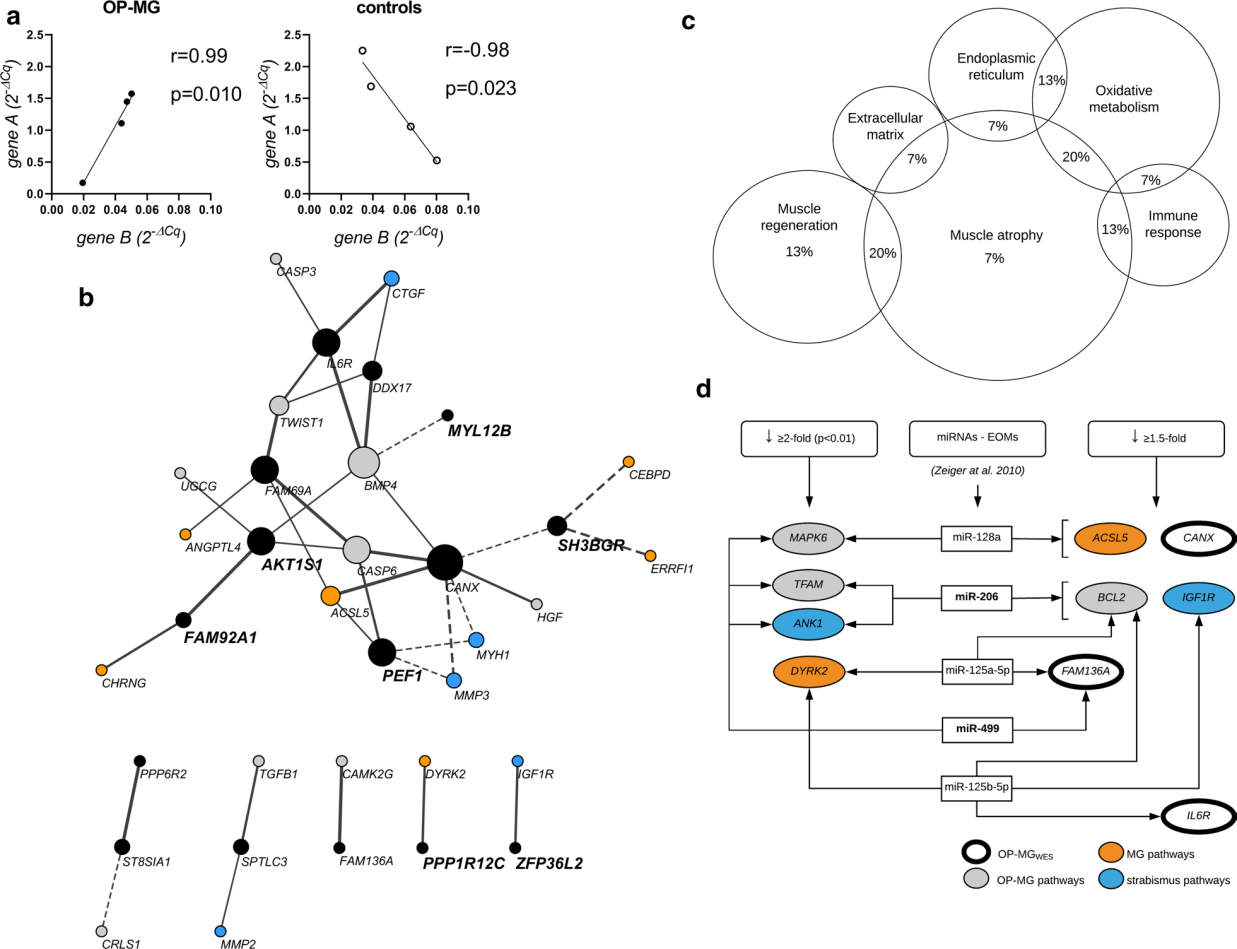
Differential and inverse co-expression of gene–gene pairs in orbicularis oculi muscles. **a** Examples of gene–gene interactions which are significantly correlated in both ophthalmoplegic myasthenia gravis (OP-MG) muscle and control muscle, either positively or negatively, but inversely (i.e. opposite directions; raw data in supplementary Fig. 1a). **b** Visualization of differentially co-expressed gene–gene pairs in orbicularis oculi muscles in the OP-MG network (*p* < 0.01). Solid lines indicate positive associations in OP-MG and dashed lines, negative association in OP-MG. The genes in bold are OP-MG genes identified in the whole genome sequencing analysis [6]. **c** A Venn diagram showing the broad biological categories represented by the most differentially correlated gene–gene transcript levels which are inverse by muscle phenotype (*p* ≤ 0.0001; supplementary Figure A). **d** Potential messenger RNA (mRNA) and microRNAs (miRNAs) regulatory networks in extraocular muscle. Differentially downregulated genes in OP-MG orbicularis oculi muscle (Fig. 2) and differentially co-expressed genes (Figs. 3, 4) are listed in the columns to show potential mRNA-miRNA interaction with miRs known to be highly expressed in extraocular muscles (EOMs). OP-MG_wes_ refers to genes identified by whole exome sequencing analysis [5]

Next, a differential co-expression algorithm was used to statistically identify gene pairs differing significantly and inversely between the two phenotypes (Fisher’s Z tests, *p* < 0.002). An example is showed in Fig. 4a with significant positive correlations in a gene pair in one phenotype (OP-MG or controls) but inverse correlations in the other (*p* < 0.01) (see Additional file 1: Figure S1). Figure 4b displays a correlation network of the significant inversely correlated gene pairs for the OP-MG network (implying that the control network showed opposite correlations). The importance of this analysis is that it shows inverse gene–gene interconnectivity (correlations) in OP-MG orbital muscle vs controls, which included seven (of nine) genes prioritized as OP-MG genes in the previous unbiased genomic comparisons viz. *PEF1*, *FAM92A1*, *AKT1*, *MYL12B*, *PPP1R12C*, *ZFP36L2* and *SH3BGR* [6]. Interestingly, the gene product of *SH3BGR* is involved with myosin heavy chain isoforms and sarcomere function and is highly expressed in EOMs.

Figure 4c depicts the representative biological categories of strongly correlated gene pairs in the OP-MG network and inverse in controls. Of the most differentially correlated gene pairs which were inverse by phenotype (n = 13; *p* ≤ 0.001), > 50% involved a gene whose transcripts were substantially altered in EAMG [17] suggesting altered MG pathways in the muscle transcriptome of OP-MG vs controls. The broad category of ‘muscle atrophy’ signalling was the most representative functional category accounting for two-thirds of the most differential and inversely correlated gene pairs by phenotype with oxidative metabolism the next most frequent category (*p* ≤ 0.001) (genes may be implicated in 2 categories).

### Downregulated genes in OP-MG orbital muscle may be regulated by microRNAs

In OOMs the significant differential downregulation of transcripts in OP-MG suggest post-transcriptional gene repression by microRNAs (miRs) binding to 3′ regulatory variants in OP-MG as a potential regulatory mechanism. Therefore, the miRTarBase database [38] was interrogated for interactions between miRs previously shown to be highly expressed in EOMs [39], and genes differentially downregulated ≥ 1.5-fold in OP-MG, compared with controls. MiR-499 and miR-206, the most highly expressed miRs in EOM (> sevenfold higher than in skeletal muscle), potentially interact with three of the genes which showed differential repression in OOMs (Fig. 4d). In addition, *CANX* and *IL6R*, which feature prominently in the differential gene–gene cross-correlations (Fig. 4b), and predicted to have EOM-miRs interactions, were prioritized previously to have 3′ regulatory region variants present in the OP-MG, but not controls [5, 7].

### Extraocular muscle

**Fig. 5:**
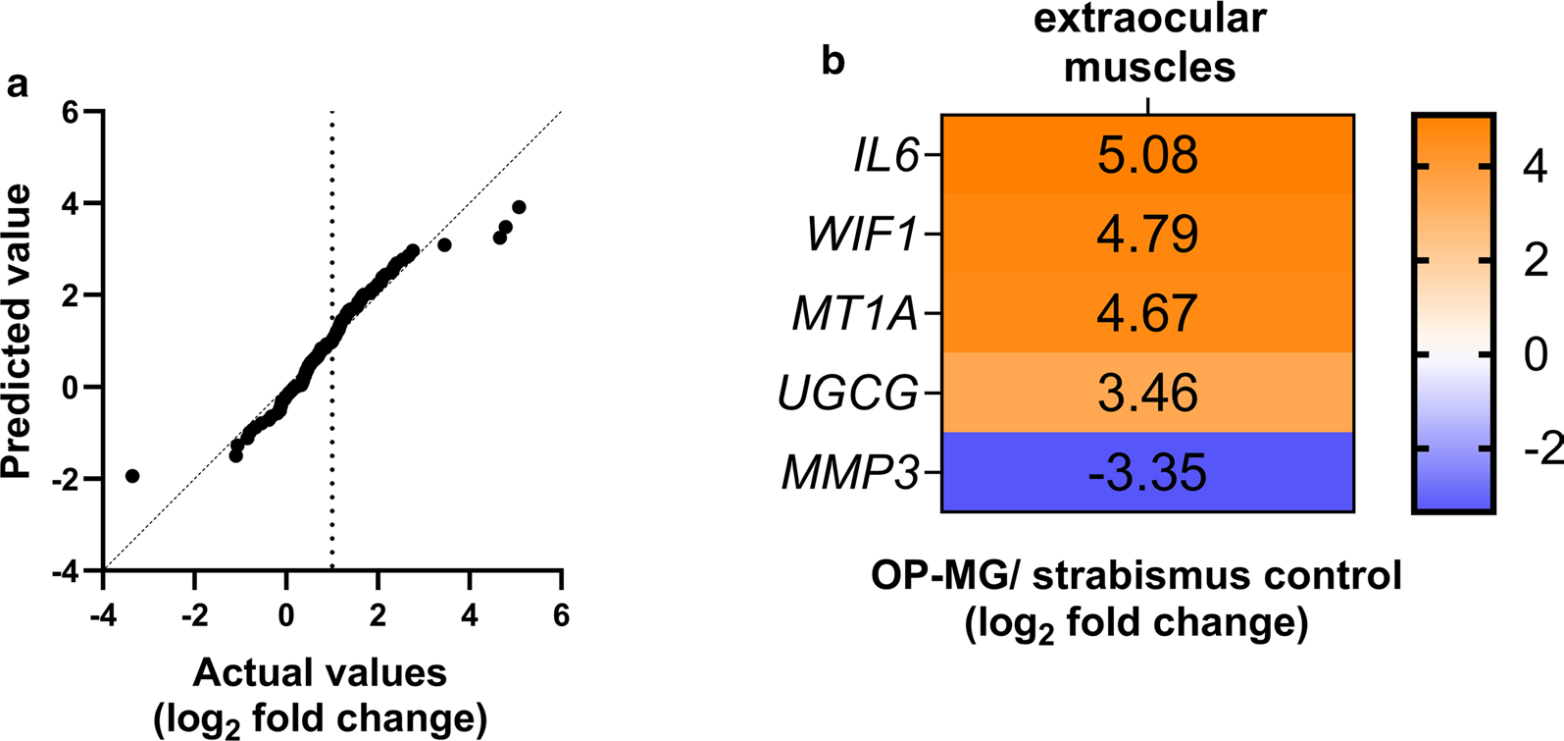
Genes differentially expressed in the medial rectus of an ophthalmoplegic myasthenia gravis (OP-MG) patient. **a** Quantile–quantile plot showing the distribution of log_2_ fold change values between the OP-MG and control extraocular muscle (medial recti) and **b** outlying values with > tenfold differences in gene expression values are represented in the heatmap

Although only two EOMs (medical recti) passed quality control we briefly mention the results due to scarcity of data on these tissues from live donors. For the EOMs, *ACTB* was used for normalization (SD = 0.06 between samples; Additional file 1: Table S2). Of relevance to the interpretation of the OOM results, the normalized expression levels of the 125 genes were similar between the two EOMs and nine OOMs (*p* = 0.72), although the EOM-specific isoform, *MYH3*, differed significantly (*p* < 0.0001; Additional file 1: Table S3, Figure S2). As the comparison of gene expression between only two samples required cautious interpretation a quantile–quantile plot of the transcripts of the two samples showed a similar distribution of the majority of the genes with only isolated values > tenfold difference (Fig. 5a, b). We briefly discuss *WIF1*, *IL6* and *MT1A* transcript levels which were ≥ 25-fold higher in OP-MG compared to control.

*WIF1* encodes Wnt inhibitory factor-1, and as an inhibitor of Wnt signalling may affect muscle regeneration and endplate AChR clustering [40]. *MT1A* encodes metallothionein-1A and protects cells against oxidative stress [in [18]]. Increased expression of metallothionein 1 has been reported in atrophic muscle, possibly by preventing the upregulation of the insulin growth factor (IGF)-1 pathway [41], and in the EOMs of the EAMG rodent models [18]. *IL6* encodes interleukin-6, a pivotal myokine in satellite cell/myoblast proliferation amongst others, but with persistently elevated levels it facilitates muscle atrophy and inhibits IGF-1 signalling [42]. Therefore, the higher transcript levels of *IL6* and *MTIA* in the chronically paralysed EOM of an OP-MG vs a non-paralytic control, could be compatible with severely atrophic muscle and/or MG-induced effects [17].

## Discussion

Gene expression profiling in orbicularis oculi muscles showed the genes which were previously prioritized as OP-MG-associated variants, were differently regulated in the muscles of OP-MG cases compared with normal controls, and supported their involvement in OP-MG pathogenesis. The original gene discovery strategy used an ‘extreme phenotype’ in which all the cases had MG, but who differed only by their EOM resistance to treatment observed in the clinic. In the current study we aimed to validate expression differences in these putative OP-MG genes/pathways using orbital muscles from affected cases vs controls without MG. Differential gene repression and gene–gene correlations differed significantly between the OOMs from OP-MG cases and control muscle. Importantly, genes such as *PEF1* and *FAM92A1*, which featured prominently in the dysregulated gene correlation networks by phenotype, were genes with unknown function when prioritized and included in the custom panel. *FAM92A1*, has recently been found to be critical for mitochondrial ultrastructural integrity [36]. Although *PEF1* remains with poorly characterised function [43], the strong correlations with the transcripts of several genes related to oxidative metabolism and extracellular matrix remodelling, suggest “guilt-by-association”. These findings are highly suggestive of perturbed crosstalk in the OP-MG orbital muscles between genes representing the muscle atrophy/regeneration and mitochondrial/oxidative metabolism functional categories.

In addition to assessing the putative OP-MG gene transcripts in orbital muscles, we were also interested in whether the transcriptional profile of genes known to represent MG/EAMG and strabismus-related pathways, may cross correlate with OP-MG gene transcripts. Here we included strabismus-related genes based on reports showing dysregulation in maligned EOMs (strabismus) in a non-MG context. However, recently RNA-sequencing of cultured human myoblasts revealed that AChR-antibodies significantly impacted genes related to extracellular matrix and actin/myosin cytoskeleton pathways [44], which overlap with the genes categorised in Fig. 1 as ‘strabismus pathways’, such as myosin genes. Although only selected myosin genes were included in the OOM profiling, they featured as dysregulated genes (Figs. 2, 4), and underscore the previous hypothesis based on clinical and histopathological reports in MG muscles, that loss of muscle contractility is critical in driving OP-MG pathogenesis [19, 45]. These results support the postulate that the EOMs of susceptible individuals, carrying gene variants in the myosin genes/pathways amongst others, are particularly vulnerable during the active MG attack to extended periods of poor contractility.

Genes related to ‘muscle atrophy signalling’ pathways at cellular level including those regulating muscle fibre size by protein synthesis (IGF1-Akt/mTOR signalling pathway), degradation (ubiquitin-proteosome and autophagy-lysosome pathways) and the balance maintained by the TGFβ-BMP superfamily [46], were common to differential gene-pair cross-correlations summarised in Fig. 4c. Interestingly, *MYH2* which showed significant repression in the OP-MG OOMs, also featured prominently in the muscle genes regulated by AChR-antibodies [44]. MYH2 is a substrate of MuRF1 and therefore directly responsive to atrophy signalling [47]. MuRF1, in turn, is moderated by the IGF1/PI3K/Akt signalling pathway which inhibits induction of muscle atrophy pathways [47]. The observed gene-pair correlations, in which transcript levels in genes from these pathways in OP-MG muscle were in direct opposite to those observed in controls, suggest perturbations between the muscle atrophy, cytoskeletal components and regeneration pathways in OP-MG OOMs. A concern for the translation of these results to the clinic is that under experimental conditions using dexamethasone, myosin heavy chain transcripts are downregulated resulting in steroid-linked muscle atrophy [47]. Steroid therapy, such as prednisone, is an important adjunct to immune therapies in MG, and in the early management of myasthenic ocular muscle paralysis, we have observed improved clinical outcomes linked with higher doses of prednisone [4]. However, it is not known whether higher doses of prednisone will benefit all individuals with ocular muscle involvement such as those carrying functional variants in high-risk pathways [6, 48, 49].

The differential gene downregulation of gene transcripts in OP-MG muscle compared to controls, implicate regulatory mechanisms mediated by miRs. Bioinformatic tools predict several miR-mRNA interactions by miRs which are highly expressed in EOMs and genes which showed differential repression in OP-MG muscle. Furthermore, OP-MG associated genes with putative functional 3′ regulatory variants, such as *DDX17* [5, 7] (Figs. 3c, 4b), featured prominently in the differential and inverse cross-correlations by phenotype in the orbital muscles, suggesting its involvement in dysregulated crosstalk. *DDX17*, which was highly expressed in both EOMs and OOMs, is a master regulator in muscle splicing events and miRNA biogenesis [34]. The study of circulating miRs as possible prognostic biomarkers for treatment-responsiveness in EOMs, may prove useful for future clinical treatment trials.

We acknowledge a number of limitations to this work. The sample sizes are limited due to opportunistic sampling of rare events. As the opportunity to evaluate the expression of putative OP-MG genes in the EOM of a well-characterized OP-MG case is a rarity, even the comparison of a single OP-MG medial rectus sample to a control was considered a valuable addition despite years of paralysis in the OP-MG EOM which would have impacted the gene-transcript snapshot. Although the study conclusions are based on the expression levels within the larger sample of OOMs and despite the similar origins of EOMs and craniofacial muscles (orbital orbicularis oculi), the EOM allotype differs from OOMs in some respects notably fatigue resistance, multi-innervated fibres (compared to singly innervated fibres in OOMs) and EOMs are constantly regenerating [12, 15]. Nevertheless, the orbital-derived orbicularis oculi muscles were not clinically weak and therefore a good comparator to the normal control muscle transcripts, and the gene transcript levels did not differ significantly between EOMs and OOMs.

## Conclusion

The profiling of patient-derived orbital muscle supports the previous unbiased, genome-driven hypotheses of candidate genes and pathways involved in the pathogenesis of OP-MG. The dysregulated gene expression in the orbital muscles of treatment-resistant ophthalmoplegic MG cases implicate pathways related to muscle contractility and mitochondrial homeostasis and strongly suggests altered extraocular muscle-specific regulatory events.

## Supplementary Information

**Additional file 1**. Supplementary Table S1 present the results of the quality of RNA extracted from the orbital muscles. Table S2 depicts the raw data informing the reference gene selection and Table S3 the genes which were highly expressed in extraocular muscle and orbicular oculi muscles. Supplementary Figure S1. Differentially co-expressed gene pairs between OP-MG and control orbicularis oculi muscle ranked by significance derived from Fisher’s Z test. Figure S2. Scatter plot of gene expression levels in two extraocular muscles and nine orbicularis oculi muscles.

## Data Availability

The datasets during and/or analysed during the current study available from the corresponding author on reasonable request.

## Abbreviations

MG: Myasthenia gravis
OP-MG: Ophthalmoplegic myasthenia gravis
EOMs: Extraocular muscles
OOMs: Orbicularis oculi muscles
EAMG: Experimental autoimmune MG
AChR: Acetylcholine receptor
RIN: RNA integrity number
qPCR: Quantitative PCR
Cq: Quantitation cycle value
SD: Standard deviation
CV: Coefficient of variation
∆: Delta
FDR: False discovery rate
