# Supplementary material for: Gene expression profiling of orbital muscles in treatment-resistant ophthalmoplegic myasthenia gravis
Source: Orphanet J Rare Dis. 2020 Dec 11;15:346. doi: 10.1186/s13023-020-01629-9 (PMC7731744; doi:10.1186/s13023-020-01629-9)
Supplement: Supplementary file 1 — Additional file 1. Supplementary Table S1 present the results of the quality of RNA extracted from the orbital muscles. Table S2 depicts the raw data informing the reference gene selection and Table S3 the genes which were highly expressed in extraocular muscle and orbicular oculi muscles. Supplementary Figure S1. Differentially co-expressed gene pairs between OP-MG and control orbicularis oculi muscle ranked by significance derived from Fisher’s Z test. Figure S2. Scatter plot of gene expression levels in two extraocular muscles and nine orbicularis oculi muscles. [file 13023_2020_1629_MOESM1_ESM.docx]

**Additional file 1:**

**Supplementary Tab****le**

**Table S1: Quality of RNA extracted from the orbital muscles**

| Tissue characteristics | | | | | | | Expression of reference genes | | Expression of target genes | |
| --- | --- | --- | --- | --- | --- | --- | --- | --- | --- | --- |
| Phenotype | Muscle | Weight (mg) | RIN | Conc. | A260/280 | A260/230 | expressed/  profiled | Mean/median Cq (±SD/IQR) | expressed/profiled | Mean/median Cq (±SD/IQR) |
| OP-MG | OOM | 7 | 7.6 | 11.3 | 1.7 | 0.9 | 5/5 | 22.4 (±3.58) | 119/120 | 27.7 (±2.63) |
| OP-MG | OOM | 3 | 7.3 | 15.6 | 1.6 | 0.4 | 5/5 | 26.8 (±2.82) | 100/120 | 31.4 (29.4: 32.6) |
| OP-MG | OOM | 8 | 6.1 | 9.3 | 1.8 | 0.8 | 5/5 | 24.0 (±3.25) | 117/120 | 28.4 (±2.54) |
| OP-MG | OOM | 6 | 6.9 | 18.6 | 1.6 | 0.5 | 5/5 | 25.5 (±3.60) | 113/120 | 29.1 (±2.35) |
| OP-MG | OOM | 6 | 6.6 | 35.3 | 1.6 | 0.4 | 5/5 | 25.3 (±3.23) | 104/120 | 30.3 (28.9: 31.8) |
| Control | OOM | 11 | 7.4 | 11.8 | 1.8 | 1.0 | 5/5 | 22.1 (±3.37) | 118/120 | 27.7 (26.2: 29.4) |
| Control | OOM | 9 | 7.0 | 18.3 | 1.7 | 0.5 | 5/5 | 23.4 (±3.73) | 119/120 | 28.5 (±2.91) |
| Control | OOM | 12 | 6.7 | 4.9 | 1.6 | 0.3 | 5/5 | 26.5 (±3.91) | 105/120 | 31.3 (29.5: 32.8) |
| Control | OOM | 26 | 5.5 | 39.7 | 1.9 | 0.7 | 5/5 | 27.4 (±3.15) | 101/120 | 29.9 (28.1: 31.1) |
| OP-MG | EOM | 21 | 5.2 | 49.0 | 1.9 | 1.6 | 5/5 | 20.1 (±3.63) | 120/120 | 24.8 (±2.68) |
| Control | EOM | 8 | 7.5 | 40.1 | 1.9 | 0.8 | 5/5 | 20.8 (±3.45) | 120/120 | 25.7 (±2.68) |

Legend: RNA integrity number (RIN); concentration (conc.); quantitation cycle (Cq). The Cq values are mean with standard deviation (SD) or median with interquartile range (IQR) depending on data distribution (Shapiro-Wilk test of normality). Only Cq values <35 are included. Ophthalmoplegic myasthenia gravis (OP-MG); extraocular muscle (EOM); orbicularis oculi muscle (OOM).

**Table S2. Reference gene selection**

|  | Average fold change OP-MG/ controls | p value | geNorm (m value) | Bestkeeper (SD; CV) |
| --- | --- | --- | --- | --- |
| **Orbicularis oculi samples (5 OP-MG; 4 control)** | | | | |
| *RPLP0* | 0,98 | 0.977 | 1.58 | 1.13; 3.78 |
| *ACTB* | 0.40 | 0.439 | 1.71 | 2.11; 8.17 |
| *GAPDH* | 0.55 | 0.565 | 2.08 | 2.41; 10.33 |
| *CSNK2A2* | 0.91 | 0.891 | 1.40 | 1.70; 7.44 |
| *ACTN2* | 1.39 | 0.559 | 1.50 | 1.05; 4.74 |
| Average *RPLP0-ACTN2* | 1.19 | 0.769 | 0.44 | 1.09; 4.19 |
| **Medial rectus samples (1 OP-MG; 1 control)** | | | | |
| *RPLP0* | 1.68 | - | 0.35 | 0.37; 1.43 |
| *ACTB* | 0.94 | - | 0.71 | 0.04; 0.21 |
| *GAPDH* | 1.41 | - | 0.40 | 0.25; 1.31 |
| *CSNK2A2* | 1.92 | - | 0.39 | 0.47; 2.70 |
| *ACTN2* | 2.77 | - | 0.67 | 0.74; 4.04 |
| Average *ACTB-GAPDH* | 1.15 | - | 0.29 | 0.10; 0.51 |
| Average *RPLP0-CSNK2A2* | 1.79 | - | 0.13 | 0.42; 1.94 |

Treatment-resistant ophthalmoplegic myasthenia gravis (OP-MG); Fold change = 2^-∆Cq^ (∆Cq=OP-MG Cq^ref^- controls Cq^ref^). “Average fold change” refers to the arithmetic mean of OP-MG 2^-Cq^ divided by the arithmetic mean of the controls. In the medial recti samples only the fold change is presented as no statistical tests could be performed on the limited number of samples. The “geNorm m value” refers to the average expression stability value calculated using the geometric mean. SD is the standard deviation and CV is the coefficient of variance.

**Table S3. Genes highly expressed in extraocular muscle (EOM) and orbicular oculi muscle (OOM)**

| Gene Symbol | Average 2^-ΔCq^ (EOM) | Average 2^-ΔCq^ (OOM) | Fold change (EOMs/OOMs) | p value |
| --- | --- | --- | --- | --- |
| *CSNK2A2* | 27,74 | 11,18 | 2,48 | 0,005 |
| *MYH2* | 16,55 | 14,70 | 1,13 | NS |
| *DES* | 15,96 | 26,51 | 0,60 | NS |
| *ACTN2* | 15,69 | 15,62 | 1,00 | NS |
| ***MYL12B*** | 11,18 | 9,96 | 1,12 | NS |
| *GAPDH* | 9,83 | 16,98 | 0,58 | NS |
| *MYH1* | 7,61 | 22,36 | 0,34 | NS |
| *MYH3* | 7,42 | 0,84 | 8,84 | 0,0001 |
| *ANKRD1* | 5,40 | 1,57 | 3,44 | 0,029 |
| *CTGF* | 2,22 | 0,83 | 2,67 | 0,007 |
| *DDX17* | 1,93 | 1,12 | 1,73 | NS |
| *ACTB* | 1,92 | 2,23 | 0,86 | NS |
| *TMBIM6* | 1,71 | 1,67 | 1,02 | NS |
| ***SH3BGR*** | 1,32 | 2,41 | 0,55 | NS |
| *BNIP3* | 1,12 | 0,85 | 1,33 | NS |
| *CHRNA1* | 1,11 | 0,11 | 10,00 | 0,036 |
| *CANX* | 0,91 | 0,42 | 2,16 | 0,033 |
| *NFKBIA* | 0,83 | 0,56 | 1,49 | NS |
| *PDK4* | 0,75 | 0,92 | 0,82 | NS |
| ***ZFP36L2*** | 0,75 | 0,71 | 1,05 | NS |
| *DAF/CD55* | 0,70 | 1,86 | 0,38 | NS |
| *CYR61* | 0,58 | 0,93 | 0,62 | NS |
| *DDR2* | 0,51 | 0,61 | 0,84 | NS |
| ***PPP1R2*** | 0,44 | 0,84 | 0,53 | NS |
| *MMP2* | 0,44 | 0,72 | 0,61 | NS |
| *GPNMB* | 0,41 | 0,73 | 0,56 | NS |
| *RGS2* | 0,34 | 0,70 | 0,49 | NS |
| *IL6* | 0,16 | 1,69 | 0,10 | NS |

Comparisons of gene expression in EOMs and OOMs is presented as fold change and p values are determined by unpaired Student’s t tests or *Mann-Whitney U test. NS refers to p>0.05. Bold refers to the OP-MG genes prioritized in (Nel et al., 20109)

**Supplementary Figures**

**Figure S1. Differentially co-expressed gene pairs between OP-MG and control orbicularis oculi muscle ranked by significance derived from Fisher’s Z test**.


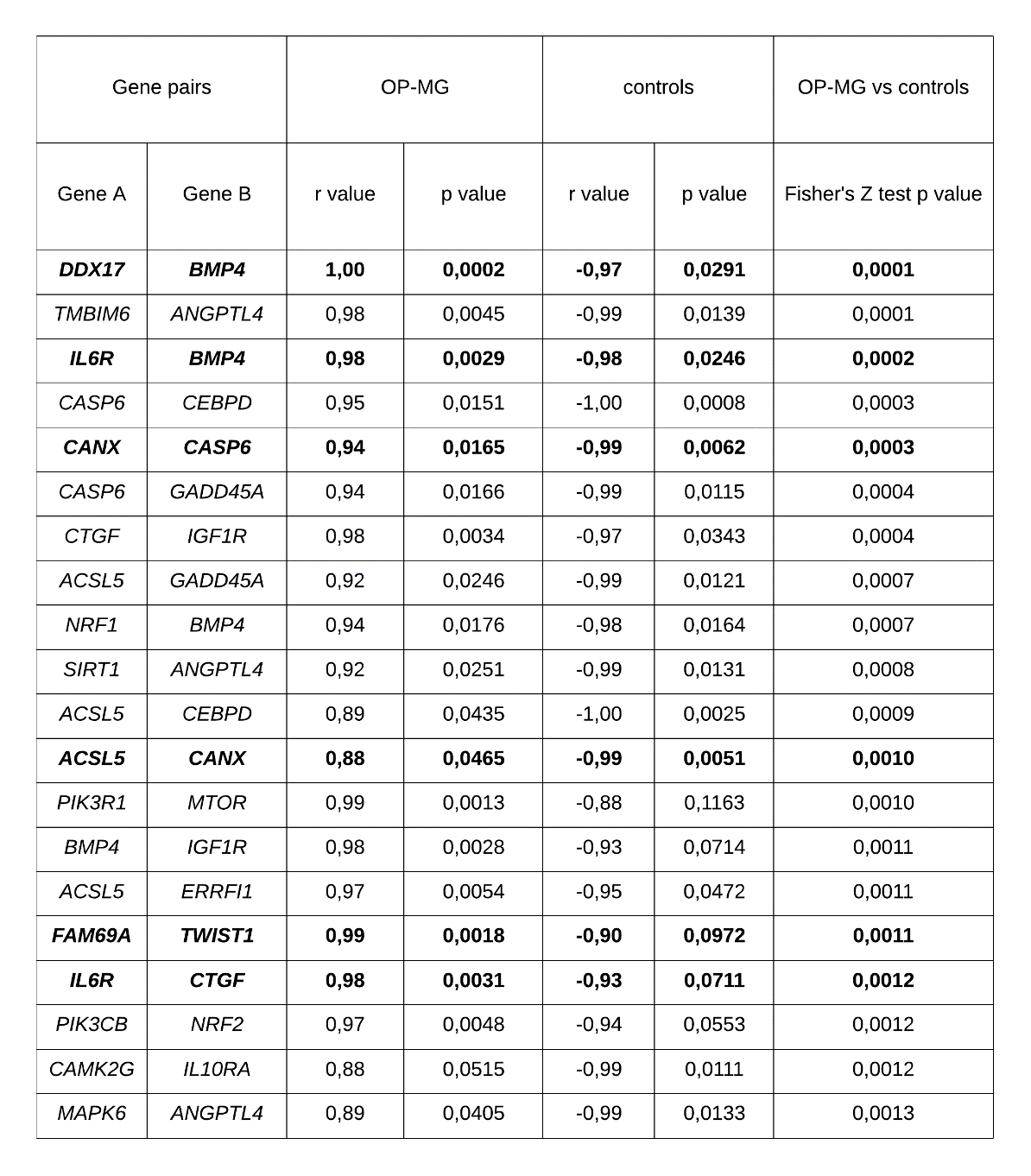


Differences between OP-MG and control correlations for each gene pair were tested for significance using the Fisher’s Z test. “r value” refers to the Pearson’s correlation co-efficient. Ophthalmoplegic myasthenia gravis (OP-MG). Gene pairs involving OP-MG genes are shown in bold text (Nel et al., 2019).

**Figure S2. Scatter plot of gene expression levels in two extraocular muscles and nine orbicularis oculi muscles.**

**
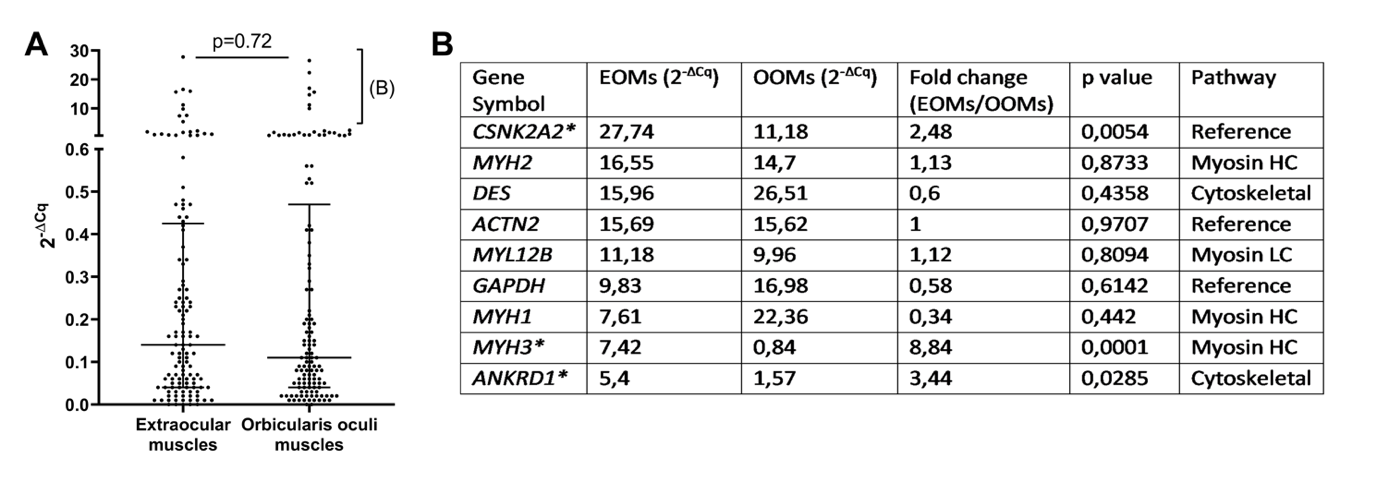
**

1. Each data point represents the average 2^-ΔCq^ value for a single gene (n=125). Error bars

indicate the median and interquartile range. After normalization, the distribution of gene expression levels for extraocular muscles (EOM) and orbicularis oculi (OOM) were similar (Mann-Whitney U test). Highly expressed genes/pathways are bracketed in (B). *indicates the gene transcripts which differed significantly between EOMs and OOMs.
